# Supplementary material for: The research on the effect of temperature of electro-surgical unit to surgical smoke distribution in theatre-in vitro and simulation study
Source: PLoS One. 2024 Mar 8;19(3):e0299369. doi: 10.1371/journal.pone.0299369 (PMC10923457; doi:10.1371/journal.pone.0299369)
Supplement: S1 Table — (DOCX) [file pone.0299369.s001.docx]

**Table 1 Thermophysical properties of materials**

| **Materials** | **Density**  **kg/m^3^** | **Viscosity**  **Pa·s** | **Heat capacity**  **J/(kg·℃)** | **Heat transfer coefficient**  **W/m·℃** |
| --- | --- | --- | --- | --- |
| **Air** | 1.225 | 1.79×10^-5^ | 1006 | 0.024 |
| **Waste gas** | 1.123 | 1.79×10^-5^ | 968 | 0.025 |
| **Non-viable particles** | 2000 | / | 1730 | 0.33 |
| **Condensed tar droplets** | 1100 | / | 1500 | 0.045 |
